# Pavement Roughness Grade Recognition Based on One-dimensional Residual Convolutional Neural Network

**DOI:** 10.3390/s23042271

**Published:** 2023-02-17

**Authors:** Juncai Xu, Xiong Yu

**Affiliations:** 1 Key Laboratory of Nondestructive Testing Technology Ministry of Education, Nanchang 400074, China; 2 Department of Civil Engineering, Case Western Reserve University, Cleveland, OH 44106, USA

**Keywords:** pavement roughness, 1/4 vehicle vibration model, white noise method: residual convolutional network

## Abstract

A pavement’s roughness seriously affects its service life and driving comfort. Considering the complexity and low accuracy of the current recognition algorithms for the roughness grade of pavements, this paper proposes a real-time pavement roughness recognition method with a lightweight residual convolutional network and time-series acceleration. Firstly, a random input pavement model is established by the white noise method, and the pavement roughness of a 1/4 vehicle vibration model is simulated to obtain the vehicle vibration response data. Then, the residual convolutional network is used to learn the deep-level information of the sample signal. The residual convolutional neural network recognizes the pavement roughness grade quickly and accurately. The experimental results show that the residual convolutional neural network has a robust feature-capturing ability for vehicle vibration signals, and the classification features can be obtained quickly. The accuracy of pavement roughness classification is as high as 98.7%, which significantly improves the accuracy and reduces the computational effort of the recognition algorithm, and is suitable for pavement roughness grade classification.

## 1. Introduction

Pavement roughness is a significant factor for vehicle travel, which has a direct impact on both vehicle smoothness and occupant comfort, as well as the quality and service life of the road [1,2]. In order to use vehicles better and ensure road traffic safety, pavement roughness recognition has theoretical research value and practical application significance [3].

Currently, the methods for determining pavement roughness grades are measurement and reverse analysis. Measurement methods include direct measurement and non-contact measurement. The direct method uses a measuring instrument to measure the pavement roughness directly [4]. However, the direct measurement method is used less frequently today because it cannot provide real-time vehicle measurements. The non-contact measurement method uses light detection and ranging (LIDAR), thermal imaging (TI), and vehicle-mounted cameras to directly extract pavement information to recognize different pavement grades [5,6,7]. Although the direct measurement method acquires a wide range of pavements, it is more costly. The inverse analysis method refers to the installation of acceleration sensors, displacement sensors, etc., on the moving vehicle to recognize the road roughness by acquiring the vehicle vibration response to different road surfaces and combining the algorithm to reverse it [8,9]. Since the acceleration sensors are easy to deploy and low-cost, and can complete the data acquisition and transmission with high accuracy, many scholars have tried to use the inverse analysis method to achieve pavement roughness detection. In 2008, Ngwangwa et al. used the displacement obtained from the simulation of a two-degrees-of-freedom model of a 1/4 car as the input of a backpropagation (BP) neural network to achieve the recognition of the road roughness grade [10,11]. Hossain et al. focused on predicting the international roughness index (IRI) of rigid pavements using an artificial neural network (ANN) model that uses climate and traffic parameters as inputs [12,13]. Ziari et al. analyzed the capability of the support-vector machine (SVM) method to predict the pavement’s future condition [14]. Xiulai Wang et al. proposed an extreme learning machine (ELM) to identify roads based on the simulation of road roughness signals [15]. These methods establish the relationship between the vehicle response and pavement roughness through neural networks, which eliminate both the work of human calibration and the work of deriving an inverse model between the vehicle response and pavement roughness. As long as the vehicle response and pavement roughness are available, the relationship between them can be established through training. After the training is completed, the road roughness can be identified from the vehicle response.

In recent years, deep learning networks have been proposed that are more convenient and give more accurate predictions than traditional methods. The recurrent neural network (RNN) is a kind of neural network for the modeling and prediction of sequence data, which can use the output of a neuron at a certain moment as the iteration input, and this also greatly improves the training performance because the parameters in the network structure are shared [16]. Due to the problem of gradient disappearance and gradient explosion in the RNN, the improved recurrent neural network known as long short-term memory (LSTM) was proposed, which can enable long-term information preservation and successfully solve the defect problem of RNN, and this has become the most popular RNN improvement method at present [17]. In addition, a variant of LSTM—the gated recurrent unit (GRU)—has a simpler structure than the LSTM network and can also solve the long-range dependency problem of the RNN network [18]. Guanqun Liang proposed a real-time recognition method for the pavement roughness grade based on LSTM and serial acceleration, which significantly reduces the computational effort of the recognition algorithm and can achieve real-time recognition [19]. Junjun Xue also proposed a recognition algorithm for the pavement roughness grade based on the GRU and vehicle vibration response [20].

As another critical model of supervised deep learning networks, convolutional neural networks (CNNs)—such as AlexNet—have received much attention from researchers for their dramatic improvement in recognition accuracy at the ImageNet image recognition challenge in 2012 [21]. As research tends to improve the performance by increasing the depth of CNNs, it has been found that the performance degrades as the depth of the network increases. Then, Kaiming He et al. proposed ResNet based on the creation of residuals to solve this problem. When the residuals module in a layer of the network is challenging to train, and no new information is learned, the residuals module only performs constant mapping, which does not lead to the phenomenon of network performance degradation [22]. The excellent performance of CNN models in extracting features has led to remarkable achievements such as image classification, face detection, and natural language processing. Based on a 2D-CNN, Jong-Hyun Jeong proposed a deep learning IRI estimation method to use anonymous vehicles and their responses measured by smartphones as road roughness sensors [23]. Since the vibration signal is one-dimensional in the time domain, the conventional CNN may lose some feature information of the original vibration signal. In addition, the signal transformation process often relies on expert experience, which may result in losing the original features [24]. Therefore, 2D-CNNs used for image processing may not be suitable for 1D signal processing. Thus, one-dimensional convolutional neural networks (1D-CNNs) are proposed to process this problem. The 1D-CNNs are similar to 2D-CNNs, in that the input are one-dimensional data and the outputs after convolution and pooling operations are also one-dimensional, and the vibration signal can be directly input into 1D-CNNs without complex signal processing [25,26]. A 1D-CNN has fewer parameters than two-dimensional convolutional neural networks, which can reduce the dependence of two-dimensional convolutional neural networks on large-scale datasets [27].

In summary, in this paper, a lightweight one-dimensional residual convolutional neural network (1D-RCNN) for pavement roughness category diagnosis is proposed by combining a one-dimensional convolutional neural network with a residual network based on vehicle response and pavement roughness simulation. We establish a complete process, including test data preprocessing, neural network model training, and testing. The main contributions are as follows:

- The 1D-RCNN uses a one-dimensional convolutional kernel as the basic computational unit, which can directly use sensor signals as inputs and be applied to pavement roughness category recognition, simplifying the model processing process.
- The residual learning mechanism introduced in the 1D-RCNN improves the training process, and the end-to-end training method improves the network feature extraction capability after introducing residual learning.
- The 1D-RCNN is a lightweight network that requires only a small amount of data to train the classifier and perform pattern recognition, which is not very demanding in terms of data volume.

The remaining sections contain the following contents: Section 2 introduces the one-dimensional convolutional neural network's principle and architecture and describes the residual learning mechanism. Section 3 introduces the principle of establishing vehicle vibration response simulation under different grades of pavement input based on a two-degrees-of-freedom 1/4 vehicle vibration model and a random input pavement model. Finally, a lightweight 1D-RCNN model for the pavement roughness grade categorization is established based on the deep learning principle and vibration response signal simulation. Section 4 constructs the vehicle vibration response datasets under different grades of pavement roughness and trains the 1D-RCNN model based on the constructed datasets. Section 5 details the effect of the 1D-RCNN on pavement roughness category recognition and compares the results with four typical classification models. Finally, Section 6 presents several essential conclusions of this research.

## 2. One-Dimensional Residual Convolutional Neural Network

### 2.1. One-Dimensional Convolutional Neural Network

**Figure 1:**
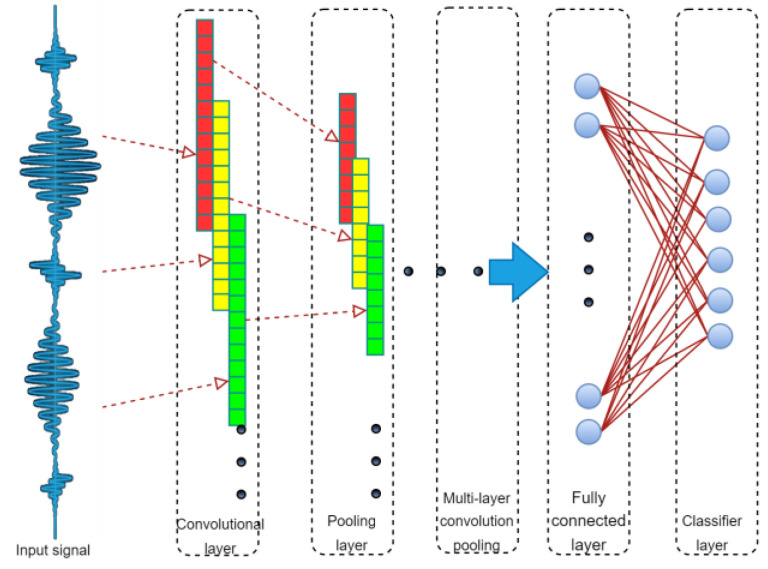
Schematic diagram of a one-dimensional convolutional neural network.

The convolutional neural network (CNN) is a special type of deep neural network. In 1984, Fukushima et al. proposed the concept of a neurocognitive machine based on the perceptual domain, which is regarded as the beginning of the formal appearance of the convolutional neural network [28]. The 1D convolutional neural network mainly consists of a 1D convolutional layer, a 1D pooling layer, a fully connected layer, and a classifier, as shown in Figure 1.

Convolution layer: for a 1D input signal $X\in R^{L}$, the convolutional layer of a 1D convolutional neural network uses *K* convolutional kernels of width *w*; $\omega_{i}\in R^{w}$ $\left(i=1,2,\cdots ,K\right)$ is a one-dimensional convolution operation, and its output is as follows:

(1) $${\;\mathrm{out}}_{\_}{\mathrm{put}}_{i}=f\left(\sum X⊙\omega_{i}+b_{i}\right)\;i=1,\cdots ,K$$

where ⊙ is the convolution operation of the convolution kernel with the input, *f* is the nonlinear activation function, *b_i_* is the bias of each channel, and *K* is the number of channels after the output.

Pooling layer: usually, the maximum pooling method for the data ***T***∈R^K*L^ is chosen, and the output after pooling is

(2) $$P_{i}(n)=\underset{0⩽n⩽\frac{L}{S}}{\max}\left\{T_{i}(nW,(n+1)W)\right\}\;i=1,\cdots ,K$$

where $T_{i}\in R^{L}$ is the *i*-th feature tensor, *W* is the size of the pooling window, and *S* is the step size.

Fully connected layer: the parameters are the weights *ω* and deviations *b*, *f* is a nonlinear activation function, and for an input *P*∈*R^m^*, the output of the fully connected layer is

(3) δ=f(ωP+b)

Classifier layer: softmax is used to obtain the label distribution of the input data:

(4) $$Q\left(\delta^{i}\right)=\frac{e^{\delta^{i}}}{\sum ke^{\delta^{k}}}$$

### 2.2. Residual Learning Network

The deeper the structure of the convolutional neural network, the higher the degree of expression and the better the fitting ability of the network. However, as the number of network layers increases, problems such as gradient explosion and gradient dispersion inevitably arise. In 2015, He et al. proposed a deep residual network model (ResNet), the essence of which is to introduce a residual module using a jump-connected network structure to superimpose shallow and deep features [22]. This effectively avoids the loss of shallow features during network training and solves the phenomenon that the network performance of the deep convolutional neural network decreases as the number of layers increases. The residual module is defined as follows:

(5) $$y=F\left(x,\left\{W_{i}\right\}\right)+x$$

where *x* and *y* are the inputs and outputs of the module, *F* denotes the residual mapping to be learned, and *W_i_* denotes the module parameters.

**Figure 2:**
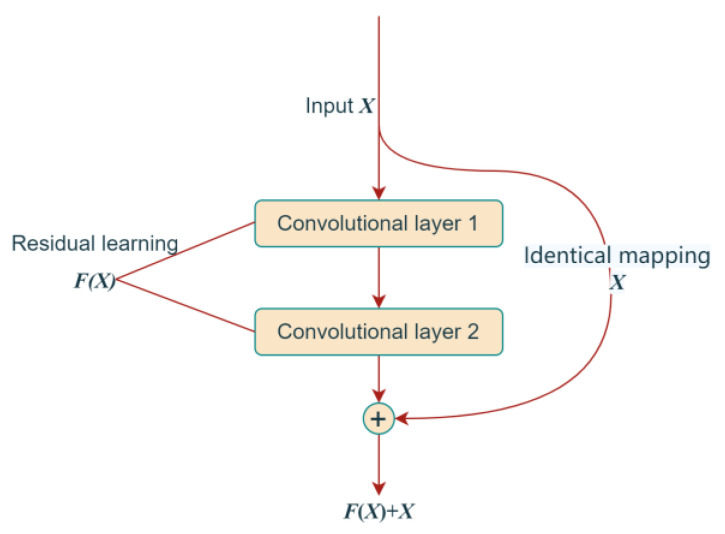
Schematic diagram of the residual module.

As shown in Figure 2, the residual learning module is no longer a potential mapping *H*(*X*) to the input of the layer, but a residual mapping *F*(*X*)* = H*(*X*) − *X*. The connection in the figure that maps the input X to the output of the module is called a jump connection, and this connection can better propagate the gradient to solve the training problem of deep convolutional neural networks while improving the network performance, as shown in Inception-V4, Res-Next, etc., which all use residual learning to improve the performance of the network. The results demonstrate that the residual learning module outperforms the baseline CNN in terms of accuracy and speed. Without the residual learning module, the CNN is limited in its ability to capture complex patterns in the data, resulting in lower accuracy and slower convergence. 

## 3. Vehicle Vibration Response under Different Roughness Grades 

### 3.1. Two-Degrees-of-Freedom 1/4 Vehicle Vibration Modeling

**Figure 3:**
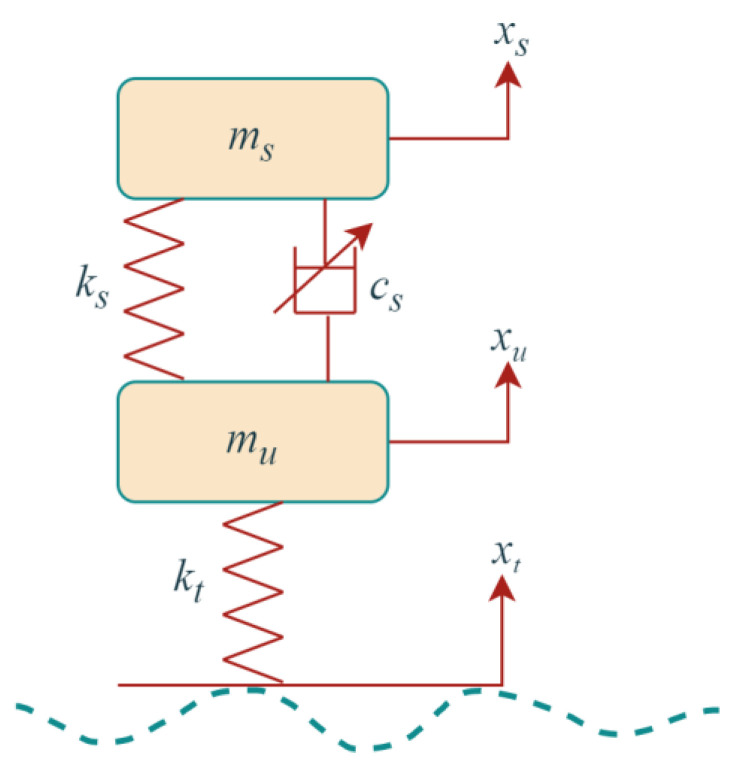
The 1/4 vehicle suspension model diagram.

The two-degrees-of-freedom 1/4 vehicle model is a simple structure that is easy to analyze and is widely used in studying the vertical dynamics of suspensions, as shown in Figure 3.

Based on Newton's second law, the kinetic differential equation is obtained as follows:

(6) $$m_{s}{\ddot{x}}_{s}+c_{s}\left({\dot{x}}_{s}-{\dot{x}}_{u}\right)+k_{s}\left(x_{s}-x_{u}\right)-u=0$$

(7) $$m_{u}{\ddot{x}}_{u}-c_{s}\left({\dot{x}}_{s}-{\dot{x}}_{u}\right)-k_{s}\left(x_{s}-x_{u}\right)+k_{t}\left(x_{u}-x_{r}\right)+u=0$$

where $m_{s},m_{u},k_{s},c_{s},k_{t},z_{s},z_{u},z_{r},u$ are the body mass, wheel mass, suspension stiffness, suspension damping, tire stiffness, body droop displacement, wheel droop displacement, road input displacement, and controlled suspension damping force, respectively.

### 3.2. Random Pavement Excitation Input Model

Usually, the pavement roughness function is defined by the change in the horizontal relative height of the pavement, the reference surface, and the road direction. The pavement roughness function is random, often considered to have a mean value of zero, and obeys a normal distribution, and the power spectral density can be used to express its characteristics. The international standard ISO 8608:2016 uses the power spectral density as a criterion for classifying pavement grades, which is expressed as follows [29]:

(8) $$G_{q}(n)=G_{q}\left(n_{0}\right){\left(\frac{n}{n_{0}}\right)}^{-\omega }$$

where n is the spatial frequency, ω is the frequency index, $n_{0}$ is the reference spatial frequency, and $G_{q}\left(n_{0}\right)$ is the pavement roughness coefficient.

The standard is based on the $G_{q}\left(n_{0}\right)$ geometric mean and the upper and lower limits of the standardized pavement classification, and Table 1 shows the range of values for category A-F pavements.

The filtered white noise method is used to generate the time-domain model of the pavement. The filtered white noise method is a method in which the ideal unit white noise is used as the input and transformed into excitation as the output after the first-order filter changes, and its expression is as follows:

(9) $${\dot{z}}_{g}(t)=-2\pi n_{1}vz_{g}(t)+2\pi n_{0}\sqrt{G_{q}\left(n_{0}\right)v}w(t)$$

where $z_{g}(t)$ is the pavement excitation, $n_{1}$ is the cutoff spatial frequency under pavement unevenness, v is the vehicle speed, and w(t) is the ideal unit of white noise with a mean value of 0 and a power spectral density of 1.

### 3.3. Establishing the Recognition Model of the Pavement Roughness Grade 

**Figure 4:**
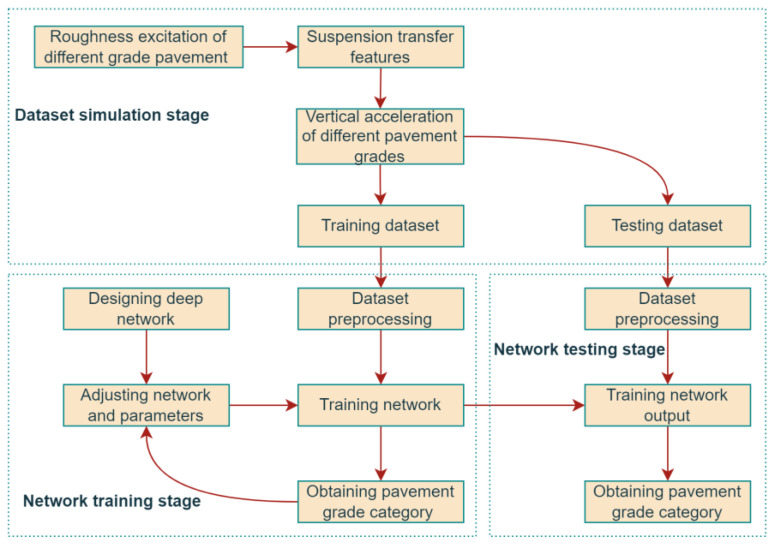
Recognition algorithm flow for the pavement roughness category.

A pavement roughness recognition algorithm can be constructed using the abovementioned deep network to capture strong features from vehicle acceleration responses. Its primary process is shown in Figure 4, which is divided into the network training and testing phases.

Deep learning requires a large amount of data for network training, and experimental data do not easily contain many complete working conditions, so simulated acceleration signals can be used as the source of the training dataset in the network training phase based on a 1/4 vehicle suspension model [30,31]. In the simulation scheme to obtain the training dataset, white noise filtering is first used to generate road excitation with different roughness grades. Then, the vehicle body’s vertical acceleration of pavements of different grades is calculated using the transmission features of the suspension. Secondly, in the simulation scheme for obtaining the dataset, acceleration sensors and data acquisition systems are assumed to be installed in the vehicle body to collect the vertical acceleration while driving on the road with different pavement roughness grades. Finally, a 10-second-long acceleration signal segment is simulated as one sample, and the above steps are repeated to merge the sampled data to form the dataset.

**Figure 5:**
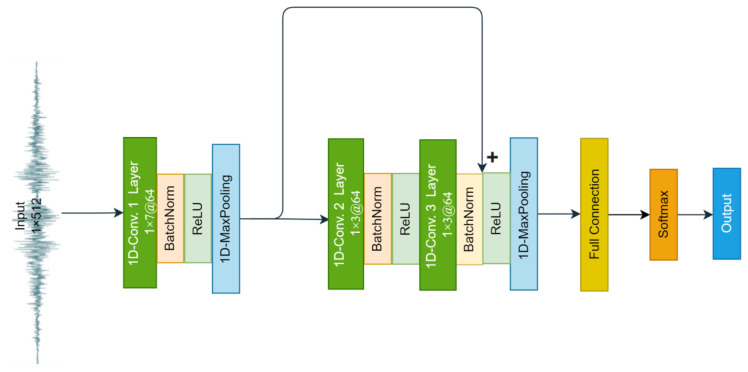
The 1D-RCNN network architecture for pavement roughness category identification.

In the training phase of the network, the 1D-RCNN network must be built (Figure 5) in the first step. Since the sequence acceleration data are directly used as inputs, layer 1 of the network is the vibration signal input layer, and each sample point in the sequence signal is input to the next layer individually. Layer 2 is the convolutional layer, where each unit is connected to the original input layer unit, and the feature extraction of the input information is initially realized through the convolutional layer. Layer 3 is the ResNet module, and the residual learning module is one core module of the whole network. The model further captures the deeper features of the upper layer’s input information and obtains the final feature information. Layer 4 is a fully connected layer, which takes the output of the previous layer of the network and performs linear operations to obtain the scores of different categories. Layer 5 is the softmax layer, which determines the probabilities of different categories and selects the largest one as the classification result.

The trained network can be applied for real-time road classification in the practical use phase. Acceleration sensors and data acquisition systems are installed on the vehicle body to collect the vertical acceleration of vehicle body while the vehicle is in motion. After intercepting the 10 s acceleration signal fragment and normalizing it, the results of pavement roughness classification can be quickly obtained by feeding it into a trained network.

## 4. Constructing Vibration Response Datasets and Training Models

### 4.1. Constructing the Datasets of the Vehicle Vibration Response

**Figure 6:**
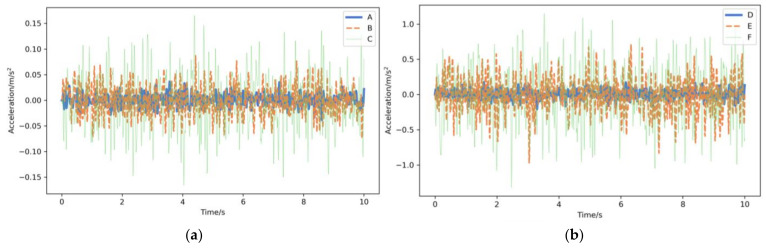
Acceleration response for different pavement roughness grades: (**a**) grades A–C; (**b**) grades D–F.

Simulation experiments were conducted using the 1/4 vehicle vibration model and the random pavement model to generate the vehicle vibration response under different pavement roughness grades. The pavement roughness simulation experiments were conducted under six pavement grades: A, B, C, D, E, and F. The simulation was with a vehicle speed of 3 m/s, a simulation time of 10 s*,* and a sampling frequency of 100 Hz (Table 2), and the vehicle response signals were obtained under all pavement grades (Figure 6). The constructed vehicle vibration response dataset contained 1800 samples and 300 samples for each pavement category. Then, the dataset was randomly divided into two groups: 70% for model training and 30% for model testing. 

### 4.2. 1D-RCNN Model Training 

The experimental environment included the Python language and PyTorch deep learning framework, along with the hardware environment of an Intel(R) Core(TM) i7-10710U CPU processor. Before conducting the experiments, the simulation dataset was normalized in order to improve the convergence speed and recognition accuracy of the pavement grade classifier. In this experiment, the Adam optimization algorithm and cross-entropy loss function were used to train the model and optimize the model fitting effect. After setting the initial parameters, the acceleration vibration signals with the corresponding pavement roughness category labels were input into the network to start the training. In the training processing, the hyperparameters (number of iterations set to 50, learning rate set to 0.001) were adjusted to achieve a high classification accuracy.

**Figure 7:**
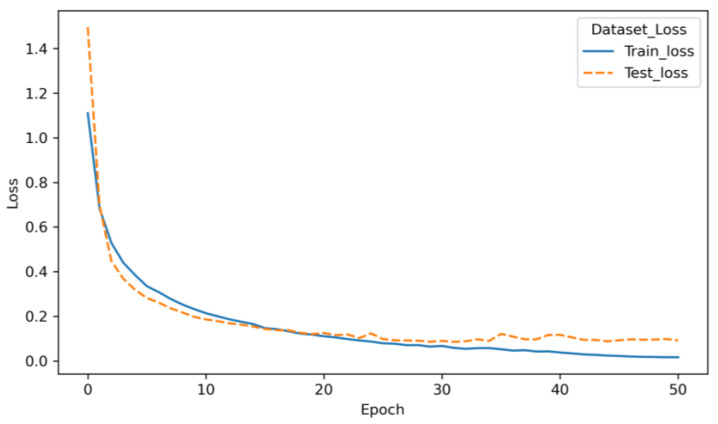
The 1D-RCNN training process loss function.

Figure 7 shows the loss function curve of the 1D-RCNN model during the training process. The loss function curve of the training set shows a decreasing trend, and the value of the loss function converges to 0 as the number of iterations increases. Meanwhile, the loss function of the testing dataset also shows a decreasing trend, so the changing trend of the loss function of the two datasets is consistent, indicating that the 1D-RCNN model does not result in overfitting and that the training effect is good. After training, the 1D-RCNN model determines the mapping relationship between the pavement roughness grade and the vehicle vibration response and establishes the recognition model.

## 5. D-RCNN Model Validation and Analysis

### 5.1. Analysis of Results

The most basic classification metric for deep learning network performance evaluation is accuracy—the ratio of the number of samples correctly classified by the classifier to the total number of samples for a given test dataset. In addition to accuracy, the three standard metrics also used to evaluate the performance of deep learning networks are precision *p*, recall *r,* and the *F*_1_-score, which are defined as follows:

(10) $$ACC=\frac{\sum TP_{i}+TN_{i}}{\sum TP_{i}+TN_{i}+FP_{i}+FN_{i}}$$

(11) $$p=\frac{TP_{i}}{TP_{i}+FP_{i}}$$

(12) $$r=\frac{TP_{i}}{TP_{i}+FN_{i}}$$

(13) $$F_{1}=\frac{2pr}{p+r}$$

where *TP* is true positive, which means that the actual is true and the prediction is also true; *FP* is false positive, which means that the actual is false and the prediction is true; *FN* is false negative, which means that the actual is true but the prediction is false; and *i* is the index of different categories.

According to Equations (10)–(13) the 1D-RCNN model evaluation was derived based on the training and test sets, and the results are shown in Table 3. According to the evaluation indices, the accuracy and recall are higher and approximately in the same pavement grade, and the *F*_1_-score is also high. The accuracy and recall rates are high for different pavement grades, indicating consistently better effects for different pavement grades. The average *F_1_*-score of 98.3% proves that the network can accurately classify different pavement roughness grades.

**Figure 8:**
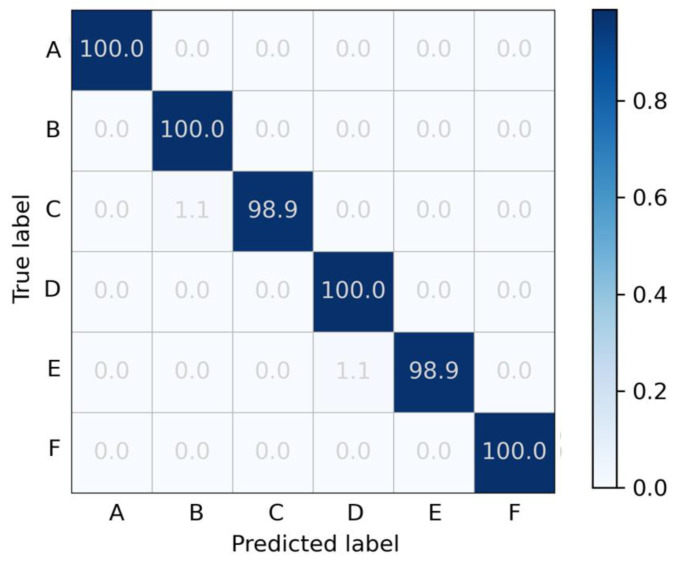
Confusion matrix of the 1D-RCNN for roughness grade classification (unit: %).

To further analyze the effectiveness of the 1D-RCNN model for classifying different types of pavements, the confusion matrix of the model for classifying six types of pavements was drawn, and the results are shown in Figure 8. The confusion matrix analysis shows that the trained network has a high accuracy rate for each type of pavement dataset. In the vast majority of cases, the predicted category is consistent with the actual category. The few incorrectly predicted pavement roughness classes are within ±1 of the actual class, with an error rate of no more than 3.3%.

**Figure 9:**
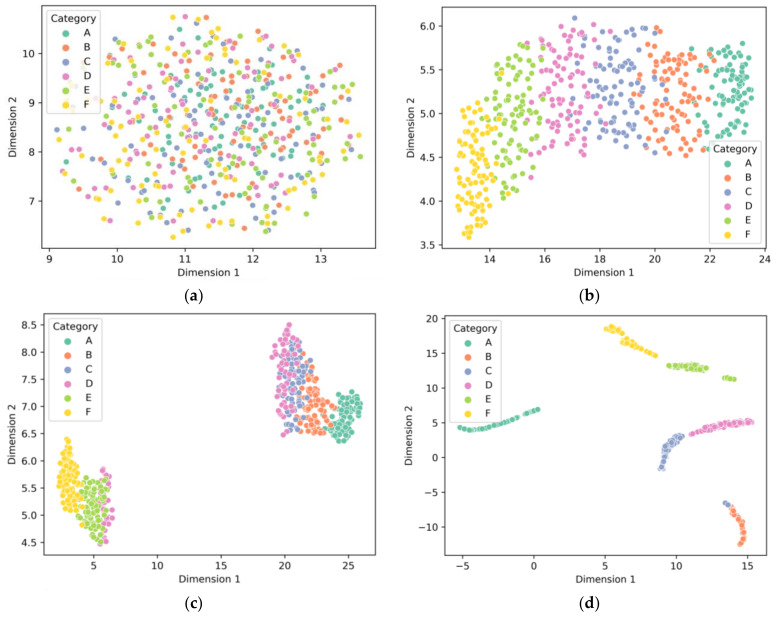
The 1D-RCNN layer output’s UMAP downscaling visualization: (**a**) first convolutional layer, (**b**) second convolutional layer, (**c**) third convolutional layer, (**d**) fully connected layer.

To exhibit the feature extraction effect of 1D-RCNN, uniform manifold approximation and projection (UMAP) is used to downscale and visualize the extracted features. Figure 9a–d represent the output after the first convolutional pooling, the output after the second convolutional pooling, the output after the third convolutional pooling, and the fully connected layer output, respectively. It can be seen that, after three layers of convolutional pooling of the output features, feature data with the same labels gradually increase the similarity of the output features in two dimensions with the convolutional layer progression. Moreover, after the convolutional pooling feature selection stage, the data with different labels achieve significant classification results in the fully connected layer.

### 5.2. Comparison of Classification Model Recognition Results

**Figure 10:**
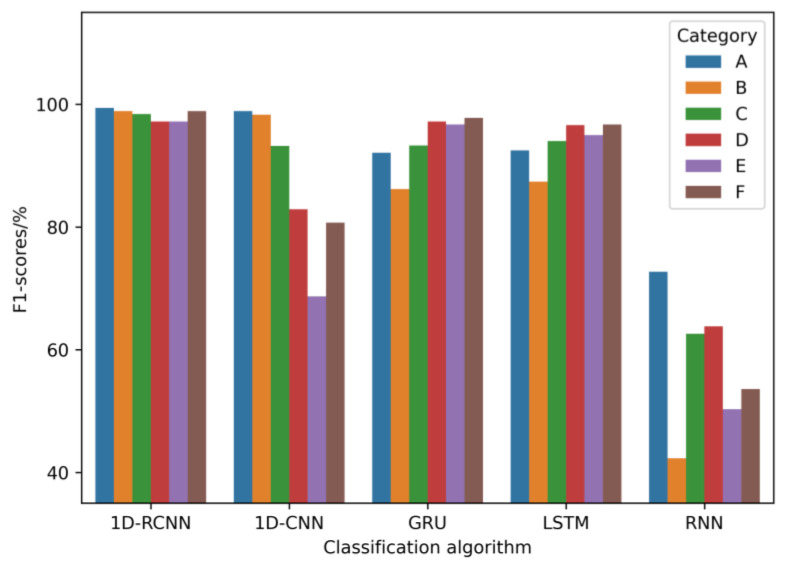
*F*_1_-score bar plots for each model’s classification recognition (unit: %).

In order to validate the proposed method, five typical models in sequence data classification were studied, and the one-dimensional convolutional network (1D-CNN), GRU (gated recurrent unit), long short-term memory (LSTM) network, and recurrent neural network (RNN) were used as the comparison models. The accuracy, precision, recall, and *F*_1_-score on the same test set were used as evaluation indices to analyze the above five models, and the comparison results are shown in Table 4. From the *F*_1_-score of each type of model, a more intuitive *F*_1_-score bar plot can be drawn, as shown in Figure 10.

From the experimental results, it can be seen that the 1D-RCNN model outperforms the other models in all evaluation metrics, achieving an accuracy of 98.4%. In addition, the GRU model's accuracy is slightly lower than that of the LSTM model. However, both are much higher than that of the 1D-CNN and RNN models. The GRU, LSTM, and 1D-CNN models used for pavement roughness classification achieve better results, but all are lower than 1D-RCNN. The performance difference between the 1D-RCNN and RNN models is significant, and the RNN models are generally effective overall. The findings of [19,20] also show that the recognition accuracy of LSTM and GRU is lower than that of the proposed 1D-RCNN, which is consistent with the results of the above analysis. The 1D-RCNN model structure uses a multilayer neural network model constructed by a 1D-CNN, which is much smaller than the common CNN models such as ResNet, VGGNet, and DenseNet in terms of computational network size and complexity. Additionally, due to its smaller scale and lower computational complexity, the sample amount required for training the model is much less than that of conventional CNN networks, and even less than the sample amount required by conventional machine learning algorithms [32].

### 5.3. Engineering Applications

**Figure 11:**
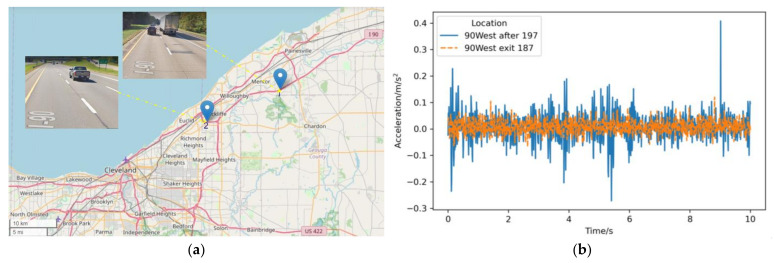
In situ vehicle vibration testing of the field pavement: (**a**) geographical location of the in situ testing; (**b**) field acquisition of raw vibration signals.

In order to further validate the practical application based on the 1D-RCNN deep learning algorithm in pavement roughness grade recognition, a road section of the I90 highway in Cleveland, Ohio, in 2005 was used as the test object. Two test locations with different roughness grades were selected for testing, where the pavement roughness grade at location 1 (90West after 197) was D, and the pavement roughness grade at location 2 (90West exit 187) was B (see Figure 11a). For this study, the vertical acceleration was measured by an ICP (integrated-circuit piezoelectric) accelerometer. The acceleration signal can be displayed in real time on a laptop. Vertical acceleration measurement system details can be found in [33]. The acceleration acquisition system acquired the acceleration vibration signals at positions 1 and 2, as shown in Figure 11b.

In the process of using the 1D-RCNN model to classify the field pavement detection, the trained weight parameters are first loaded into the 1D-RCNN model, and then the acceleration vibration signals collected at location 1 and location 2 are used as input data. The 1D-RCNN model and the output can extract the signal features of different categories. The output results at positions 1 and 2 were [−23.6449, −10.0262, 1.0111, 2.3127, −2.4176, −9.0892] and [−5.7164, 1.4103, −1.1854, −6.6368, −6.3494, −7.3417], respectively. Based on the output, the index corresponding to the maximum at location 1 is 3, while the index corresponding to the maximum at location 2 is 1. The pavement roughness classes at the measurement points are class D and class B, respectively, corresponding to the actual pavement roughness classes. From the present-day photos of the pavement, there are obvious repair traces at location 1 compared to the pavement at location 2, which also verifies that the model established in this paper can effectively identify the grades of pavements with different roughness.

## 6. Conclusions

This paper proposes a lightweight 1D-RCNN model for pavement roughness recognition by combining a one-dimensional convolutional depth network with a residual module. The trained network was tested, and the results show that recognizing the pavement roughness grade from the acceleration signal is effective and feasible. Its accuracy is over 98%, and the performance evaluation indices are all high. From the above study, the main conclusions are as follows:

1. The input is a sequence acceleration signal and does not need a frequency domain to transform, such as a Fourier transform, with no feature extraction. Instead, it directly inputs the original signal to train a deep neural network end-to-end, simplifying the model processing.
2. The proposed 1D-RCNN is based on the mass acceleration of the vehicle body in the vertical direction as the input signal, which is a single-channel input with a simple sensor arrangement and low hardware cost.
3. The 1D-RCNN uses a multilayer one-dimensional convolutional kernel and a residual learning mechanism to effectively extract the key features of the vibration signal, thereby improving the performance of the recognizer.
4. The proposed lightweight 1D-RCNN is more practical than conventional deep learning algorithms that do not require a large amount of labeled data. Moreover, the good feature learning capability makes it widely applicable for vibration signal recognition.

Since all of the training datasets used in this paper are composed of the simulation data of the vehicle vibration response, which still have some differences with in situ vehicle vibration signals, future work on some field-collected vehicle vibration signals as training data may be appropriate in order to further improve the applicability of the model. Meanwhile, due to the low cost and easy installation of acceleration sensors, data can be acquired with high accuracy, and even the current smartphones come with acceleration sensors. Furthermore, the means of transportation can be a car, truck, or bicycle, so the proposed method can be easily transferred to other carriers for application. Thus, the proposed 1D-RCNN still has some room for improvement. Further research should focus on developing more sophisticated deep learning models that can accurately recognize pavement roughness grades from various road conditions. Additionally, the research could include developing more efficient and reliable methods for collecting pavement roughness grade data, as well as applying deep learning to the analysis of such data.

## Figures and Tables

**Table 1:** Classification of pavement grades based on $G_{q}\left(n\right)$.

Grade	A	B	C	D	E	F
Upper limit	8	32	128	512	2048	8192
Geometric mean	16	64	256	1024	4096	16,138
Lower limit	32	128	512	2048	8192	32,768

**Table 2:** Vehicle model parameters.

Vehicle Parameters	Values	Vehicle Parameters	Values
Mass/kg	300	Wheel mass/kg	30
Suspension stiffness/(N.m^−1^)	10,000	Tire stiffness/(N.m^−1^)	180,000
Suspension damping/(N.s.m^−1^)	1500		

**Table 3:** Evaluation results of the training and test sets (unit: %).

Pavement Grade	Training Dataset			Testing Dataset		
	*p*	*r*	*F*_1_	*p*	*r*	*F*_1_
**A**	100	98.6	99.3	100	98.9	99.4
**B**	98.6	100	99.3	97.8	100	98.9
**C**	100	99.1	99.5	100	96.8	98.4
**D**	99.0	100	99.5	97.8	96.7	97.2
**E**	100	100	100	96.7	97.8	97.2
**F**	100	100	100	97.8	100	98.9
***ACC***	99.6			98.4		

**Table 4:** Test results of five typical deep learning classification models (unit: %).

Pavement Grade	1D-RCNN			1D-CNN			GRU			LSTM			RNN		
	*p*	*r*	*F*_1_	*p*	*r*	*F*_1_	*p*	*r*	*F*_1_	*p*	*r*	*F*_1_	*p*	*r*	*F*_1_
**A**	100	98.9	99.4	100	97.8	98.9	96.7	87.9	92.1	95.6	89.6	92.5	71.1	74.4	72.7
**B**	97.8	100	98.9	97.8	98.9	98.3	83.3	89.3	86.2	84.4	90.5	87.4	36.7	50.0	42.3
**C**	100	96.8	98.4	98.9	88.1	93.2	93.3	93.3	93.3	95.6	92.5	94.0	73.3	54.5	62.6
**D**	97.8	96.7	97.2	83.3	82.4	82.9	95.6	98.9	97.2	94.4	98.8	96.6	65.6	62.1	63.8
**E**	96.7	97.8	97.2	63.3	75.0	68.7	96.7	96.7	96.7	94.4	95.5	95.0	55.6	45.9	50.3
**F**	97.8	100	98.9	81.1	80.2	80.7	97.8	97.8	97.8	97.8	95.7	96.7	45.6	65.1	53.6
***ACC***	98.4			87.1			93.9			93.7			58.7		

## Data Availability

Not applicable.
